# Esculin improves dyslipidemia, inflammation and renal damage in streptozotocin-induced diabetic rats

**DOI:** 10.1186/s12906-015-0817-y

**Published:** 2015-11-09

**Authors:** Yue-Hua Wang, Yan-Hong Liu, Guo-Rong He, Yang Lv, Guan-Hua Du

**Affiliations:** Beijing Key Laboratory of Drug Target Identification, Institute of Materia Medica, Chinese Academy of Medical Sciences & Peking Union Medical College, Beijing, 100050 China; State Key Laboratory of Bioactive Substance and Function of Natural Medicines, Institute of Materia Medica, Chinese Academy of Medical Sciences & Peking Union Medical College, Beijing, 100050 China; China Pharmaceutical University, Nanjing, 210009 China; Beijing Key Laboratory of Drug Crystal Research, Institute of Materia Medica, Chinese Academy of Medical Sciences & Peking Union Medical College, Beijing, 100050 China

**Keywords:** Esculin, Diabetic complications, Dyslipidemia, Advanced glycation end products (AGEs)

## Abstract

**Background:**

Increasing studies have shown that dyslipidemia and inflammatory responses play important roles in the progression of microvascular diabetic complications. Esculin (ES), a coumarin derivative, was extracted from *Fraxinus rhynchophylla*. The present study was to evaluate the potential effects of ES on lipid metabolism, inflammation responses and renal damage in streptozotocin (STZ)-induced experimental diabetic rats and explore the possible mechanism.

**Methods:**

Diabetic rat model was established by administration high-glucose-fat diet and intraperitoneal injection of STZ 45 mg/kg. ES was administrated to diabetic rats intragastrically at 10, 30 and 90 mg/kg for 10 weeks respectively. The levels of triglycerides (TG), total cholesterol (T-CHO), low density lipoproteins (LDL), and high-density-cholesterol (HDL-C) in serum were measured. IL-1, IL-6, ICAM-1, NO, NAGL, and AGEs level in serum were detected by ELISA assay. The accumulation of AGEs in kidney tissue was examined by immunohistochemistry assay.

**Results:**

The results showed that ES could decrease TG, T-CHO, LDL levels in serum of diabetic rats in a dose dependent manner. ES also decreased IL-1, IL-6, ICAM-1, NO and NGAL levels in serum of diabetic rats in a dose dependent manner. Furthermore, ES at 30 and 90 mg/kg significantly decreased AGEs level in serum and alleviated AGEs accumulation in renal in diabetic rats.

**Conclusions:**

Our findings indicate that ES could improve dyslipidemia, inflammation responses, renal damage in STZ-induced diabetic rats and the possible mechanism might be associated with the inhibition of AGEs formation.

## Background

Diabetes mellitus is characterized by hyperglycaemia, hypercholesterolemia and hypertriglyceridemia, resulting from defects in insulin secretion followed by dysfunction and failure of organs especially the eyes, kidneys, nerves, heart and arteries [1]. Abnormalities in lipid profile are one of the most common complications in diabetes mellitus [2]. The key components of diabetic dyslipidemia are elevated plasma low density lipoproteins (LDL), triglycerides (TGs), and lowered high density lipoprotein (HDL-C) [3]. Several studies have also demonstrated that inflammatory processes play a crucial role in the development of diabetic complications. In the present study, in order to verify the effects of ES on anti-diabetic complications, we investigated the level of proinflammatory cytokines in the diabetic rats.

Diabetic nephropathy is a major microvascular complication of diabetes mellitus and the most common cause of renal failure. The renal injury in diabetic nephropathy is due to a series of complex pathophysiological changes such as glomerulosclerosis, vascular diseases and changes of the tubulointerstitium with tubular atrophy and interstitial fibrosis initiated by disturbed glucose homeostasis [4]. Neutrophil gelatinase-associated lipocalin (NGAL), a 25 kDa protein belonging to the lipocalin superfamily, was initially found in activated neutrophils. However, many other cells, like kidney tubular cells, may also produce NGAL in response to various insults [5]. Both serum and uric NGAL have been found to be reliable predictors of kidney injury [6].

Recent studies have suggested that advanced glycation end products (AGEs) are believed to play a crucial role in the pathogenesis of diabetic complications [7]. AGEs ligate to the receptor of AGEs (RAGE), facilitating activation of the transcription factor NFκB may be involved in the development of diabetic complications [8].

**Fig. 1:**
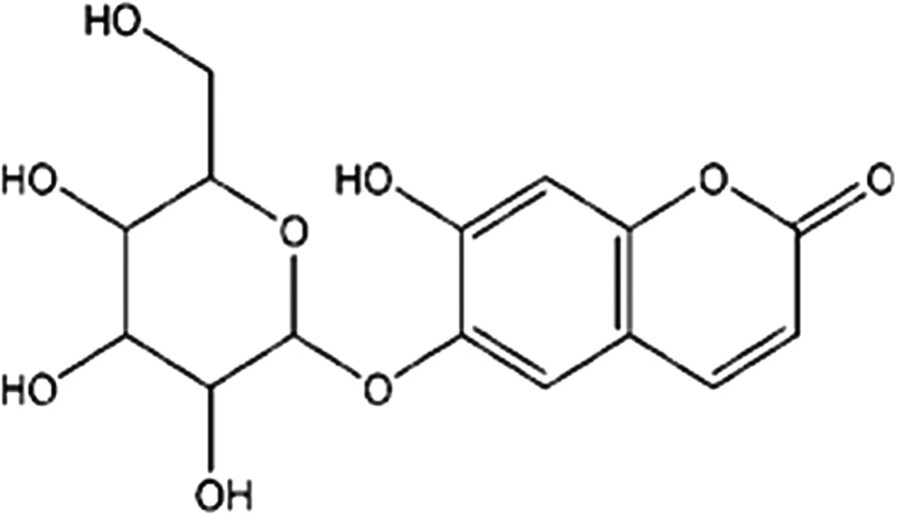
The chemical structure of esculin

Esculin (Fig. 1), a coumarin derivative, was extracted from *Fraxinus rhynchophylla*, belonging to the Oleaceae family, which is widely distributed in the east of Asia, especially in the south of China. In diabetes-related studies, it has been demonstrated that esculin and its metabolite esculetin inhibit formation of advanced glycation end-products formation [9], inhibit the activities of α-glucosidase and protein tyrosine phosphatase 1B [10], and have the beneficial effect against diabetes and oxidative stress-related inflammatory processes in the kidney of mice [11]. However, studies on the effect and mechanism of esculin on diabetic rats are limited. Therefore, we investigated the effect and possible mechanism of esculin against dyslipidemia, inflammation responses and renal damage in high-glucose-fat diet and STZ-induced diabetic rats.

## Methods

### Reagents

Esculin was purchased from Nanjing Zelang Medical Technology Co., Ltd (Nanjing, China). Streptozotocin was purchased from Sigma-Aldrich Co. (St. Louis, MO, USA). Triglyceride, total cholesterol, LDL cholesterol and HDL cholesterol were assayed using kits from Applygen Coporation (Beijing, China). One touch gluco-meter (Accu-chek sensor) was Roche products. IL-1 and IL-6 ELISA kits were purchased from ExCell Bio (Genetimes Technology, Inc., Beijing, China), ICAM-1 and NAGL ELISA Kits were purchase from Wuhan Boster Bio-engineering limited company (Wuhan, China). NO level was assayed by Griess Regant System from Promega Corporation (Madison, USA). AGEs ELISA Kit was purchase from CELL BIOLABS INC. (San Diego, CA). High-sugar-fat chow was added 10 % sugar, 10 % lard, 2 % cholesterol, 0.2 % cholic acid in the standard of rat chow.

### Animals

Adult male Sprague–Dawley rats (200–220 g) were purchased from the Animal Centre of Beijing Vitra Liver (Beijing, China). All animals were housed in a room with controlled temperature of 23 ± 2 °C and humidity of 55 ± 5 % with a regular 12 h light–dark cycle and allowed to have water and food ad libitum. All animal care and experimental procedures regarding the animals were approved by the ethic committees of Institute of Materia Medica, Chinese Academy of Medical Sciences & Peking Union Medical College.

#### STZ-induced experimental diabetic rats and experimental design

Diabetes was induced in all the rats except a group of ten animals which were treated as normal control group by intraperitoneal administration of STZ (45 mg/kg) dissolved in freshly prepared citrate buffer (pH 4.5). The animals were fasted for 12 h before STZ administration and blood glucose was estimated after 72 hours of STZ administration. The animals with blood glucose more than 16.7 mmol/l were treated as diabetic and the animals were divided into 6 groups (*n* = 10): one group was served as diabetic control; ES-L, ES-M, and ES-H groups were received esculin 10 mg/kg, 30 mg/kg and 90 mg/kg by per oral administration respectively for a period of 10 weeks; AG group was served as positive group by per oral administration. After administration, rats were anesthetized and killed, serum was collected. The kidney was separated and fixed in 4 % polyformaldehyde for histopathology assay and immunochemistry staining.

#### Determination of lipid profile in serum

Triglyceride (TG), Total cholesterol (TC), low density lipoprotein (LDL), high density lipoprotein (HDL) content in serum were assayed following the manufacturer’s protocol.

#### Determination of IL-1, IL-6, ICAM-1, NO, AGEs, and NAGL level in serum

IL-1, IL-6, ICAM-1, NO, AGEs, and NAGL level in serum were measured according to the manufacturer’s protocol.

#### Histological examination

After fixation, renal tissues were stained with hematoxylin and eosin (HE) reagents for histological examination and examined under the microscope.

#### AGEs level estimated in renal by immunochemistry

AGEs levels in renal were determined by immunochemistry staining on 4 % polyformalin-fixed paraffin sections (2-μm-thick). Briefly, the deparaffinized and rehydrated tissue sections were incubated with 3 % hydrogen peroxide to block endogenous peroxidase and then subjected to antigen retrieval through microwave oven heating in 0.1 M sodium citrate (pH 6.0) for 10 min. After that, tissue sections were incubated with 10 % normal goat serum for 10 min followed by an overnight incubation with anti-AGEs (1:100) antibody in 10 % normal goat serum at 4 °C. The sections were washed, further incubated with horseradish peroxidase (HRP)-labeled goat anti-rabbit/mouse polyclonal antibody for 20 min at 37 °C, and developed with 3,3-diaminobenzidine (DAB, Sigma) for color reaction. The sections were then counterstained with hematoxylin. All measurements were performed blindly.

#### Statistical analysis

All data are expressed as the mean ± SD. Statistical analysis of the results was carried out by one-way analysis of variance (ANOVA), followed by *t*-test. Statistical significance was accepted at *P* < 0.05.

## Results

### Effect of ES on body weight and blood glucose in STZ-induced diabetic rats

**Fig. 2:**
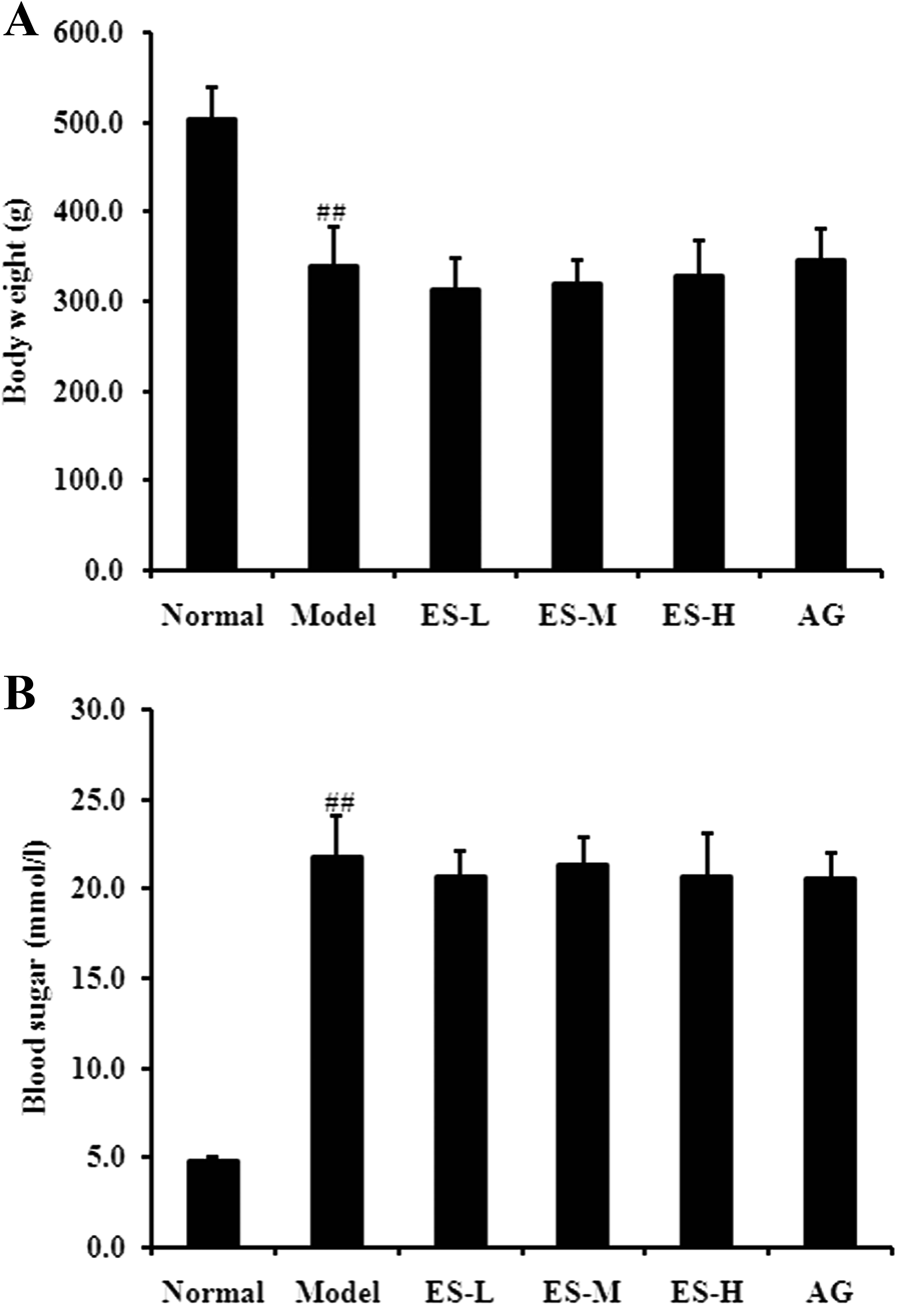
Effects of ES on body weight and blood sugar in STZ-induced diabetic rats. **a** Body weight; **b** Blood sugar. Data are expressed as means ± S.D. (*n* = 6 ~ 8). ^##^ *P* < 0.01 vs normal control group; ^*^ *P* < 0.05, ^**^ *P* < 0.01 vs diabetic model group

The diabetic model rats exhibited reduced body weight and increased blood glucose levels compared with normal control rats (both *P* < 0.01). However, there were no effect on body weight (Fig. 2a) and blood glucose (Fig. 2b) observed in ES or AG-treated rats.

### Effect of ES on biochemical parameters in STZ-induced diabetic rats

**Fig. 3:**
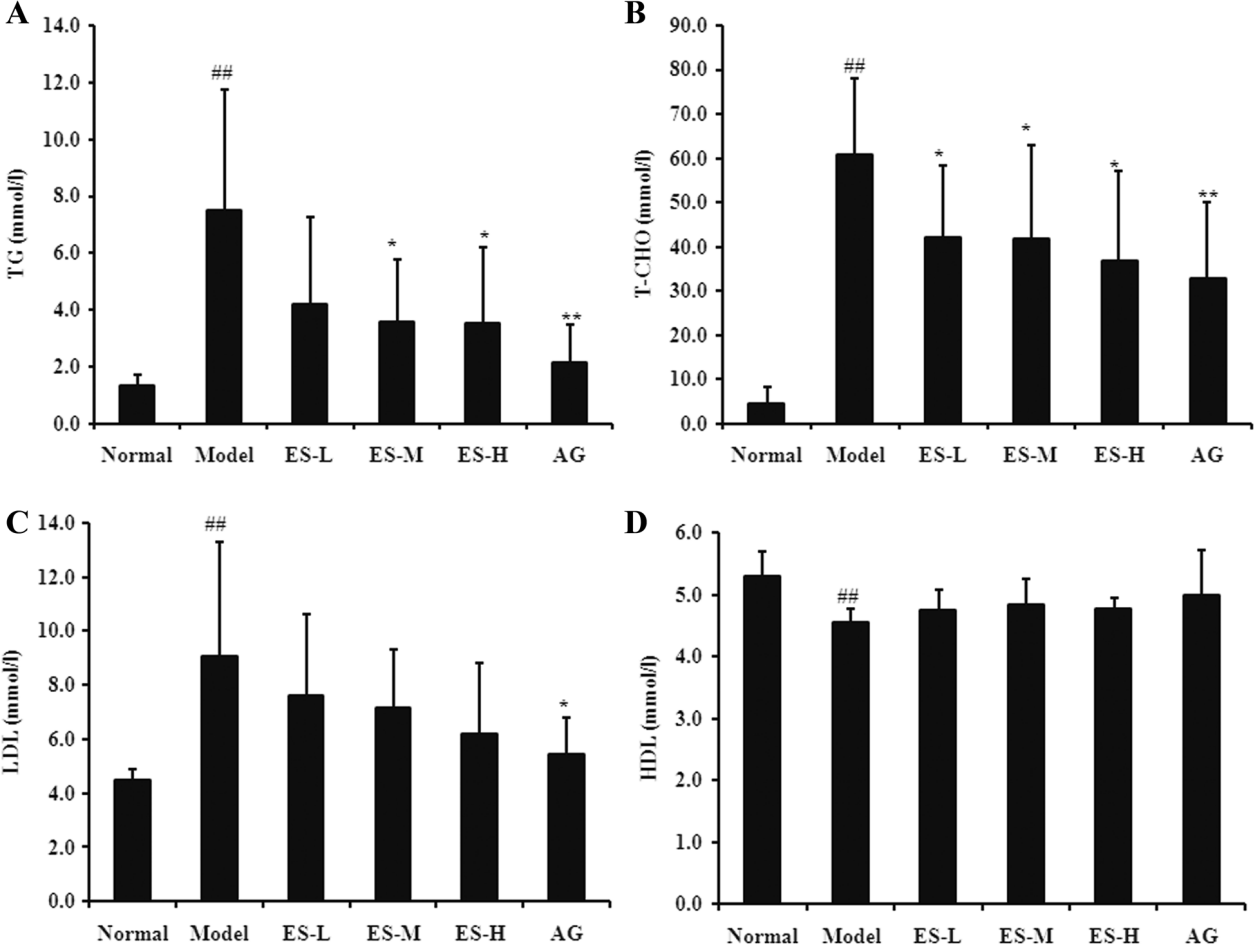
Effect of ES on TG, T-CHO, LDL, and HDL levels in STZ-induced diabetic rats. **a** TG level; **b** T-CHO level; **c** LDL level; **d** HDL level. Data are expressed as means ± S.D (*n* = 6 ~ 8). Data are expressed as means ± S.D. (*n* = 6 ~ 8). ^##^ *P* < 0.01 vs normal control group; ^*^ *P* < 0.05, ^**^ *P* < 0.01 vs diabetic model group

In diabetic control rats, TG, T-CHO, and LDL levels were all significantly increased when compared to normal rats (all *P* < 0.01). However, treatment with ES dose-dependently attenuated the increase in TG, T-CHO, and LDL from the diabetic rats (Fig. 3a, b, and c), but these levels were still higher than that observed in normal rats and AG-treated rats. These findings indicate that ES is able to ameliorate diabetes signs to a certain extent.

Rats in the diabetic model group demonstrated significant decrease of HDL compared to the control group (*P* < 0.01). However, after treatment with ES, there was no significant change in HDL level all of these parameters improved significantly. Similar significant improvements in these parameters occurred in the AG group as well (Fig. 3d).

### Effect of ES on inflammatory factors in STZ-induced diabetic rats

**Fig. 4:**
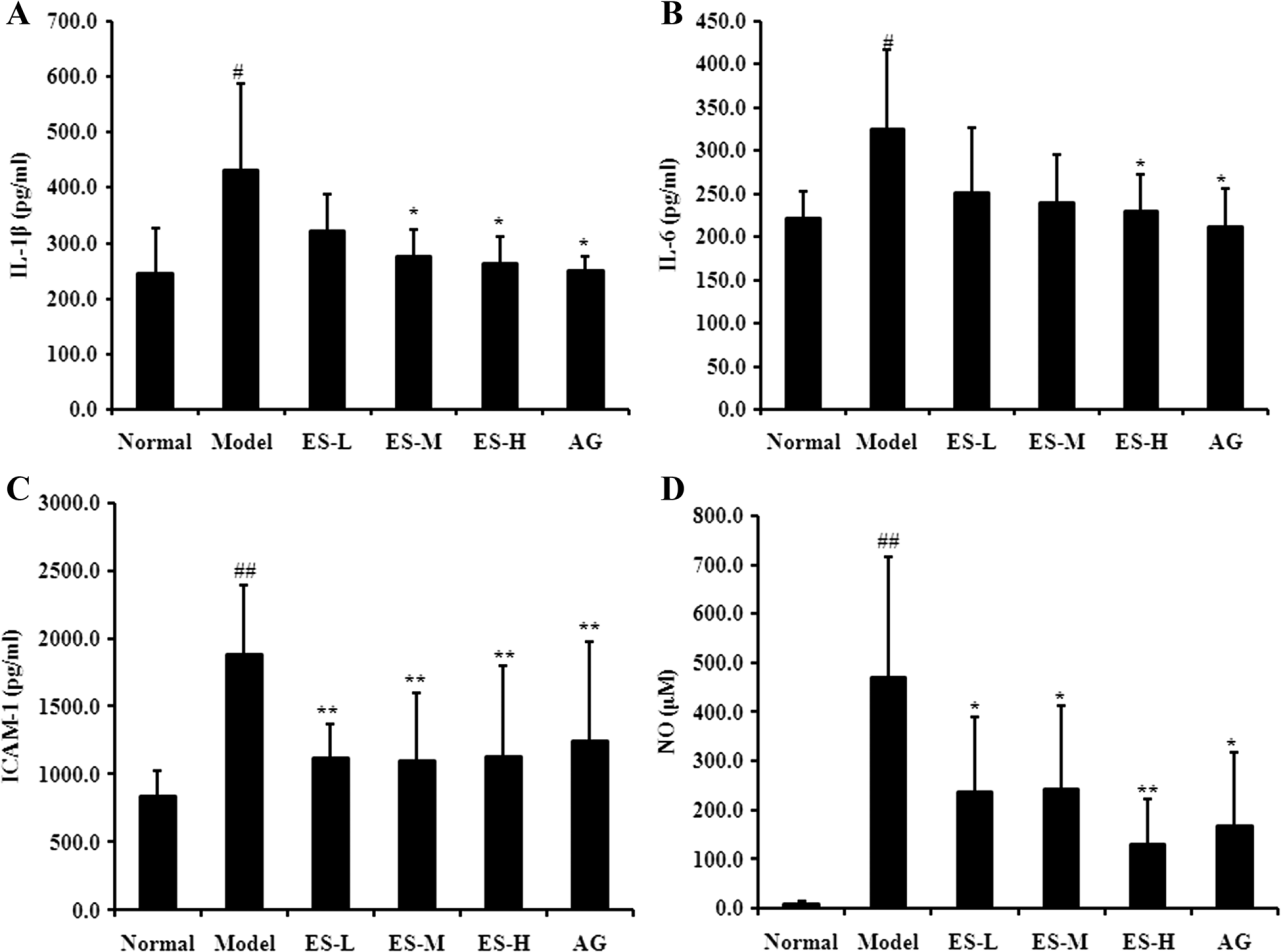
Effect of ES on inflammation responses in STZ-induced diabetic rats. **a** IL-1 level; **b** IL-6 level; **c** ICAM-1 level; **d** NO level. Data are expressed as means ± S.D. (*n* = 6 ~ 8). Data are expressed as means ± S.D. (*n* = 6 ~ 8). ^##^ *P* < 0.01 vs control group; ^*^ *P* < 0.05, ^**^ *P* < 0.01 vs diabetic model group

In diabetic model rats, IL-1, IL-6, ICAM-1 and NO levels were all significantly increased compared with normal control rats (*P* < 0.05, *P* < 0.05, *P* < 0.01, *P* < 0.01, respectively). However, treatment with ES dose-dependently attenuated the increase in IL-1, IL-6, ICAM-1 and NO levels from the diabetic rats (Fig. 4). These findings indicate that ES is able to ameliorate inflammatory responses to a certain extent in diabetic rats.

### Effect of ES on NAGL level in serum in STZ-induced diabetic rats

**Fig. 5:**
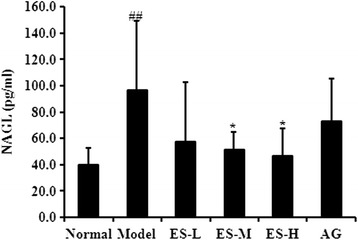
Effect of ES on NAGL level in STZ-induced diabetic rats. Data are expressed as means ± S.D. (*n* = 6 ~ 8). Data are expressed as means ± S.D. (*n* = 6 ~ 8). ^##^ *P* < 0.01 vs control group; ^*^ *P* < 0.05, ^**^ *P* < 0.01 vs diabetic model group

As shown in Fig. 5, the NAGL level in serum was significantly increased in STZ-induced diabetic rats compared with the normal control rats (*P* < 0.01). However, treatment with ES 30 and 90 mg/kg significantly decreased the NAGL levels compared with the diabetic model group (both *P* < 0.05), and the level was lower than that observed in AG-treated rats (Fig. 5). These results indicate that to some extent, ES is able to protect against diabetic kidney injury.

### Effect of ES on AGEs level in serum in STZ-induced diabetic rats

**Fig. 6:**
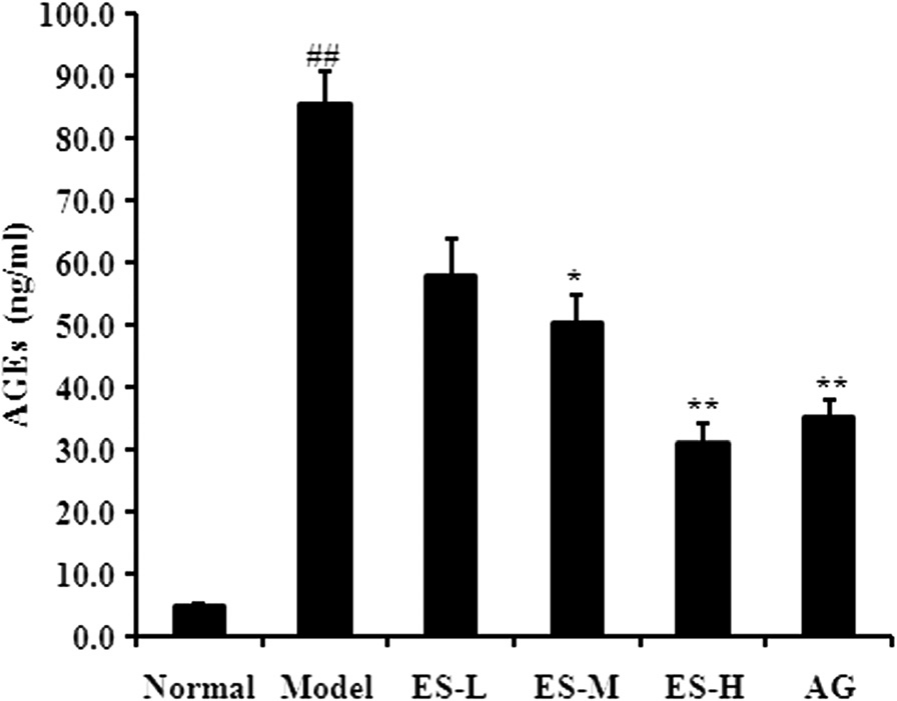
Effect of ES on AGEs level in STZ-induced diabetic rats. Data are expressed as means ± S.D. (*n* = 6 ~ 8). Data are expressed as means ± S.D. (*n* = 6 ~ 8). ^##^ *P* < 0.01 vs control group; ^*^ *P* < 0.05, ^**^ *P* < 0.01 vs diabetic model group

As shown in Fig. 6, the AGEs level in serum was significantly increased in STZ-induced diabetic rats compared with the normal control rats (*P* < 0.01). In contrast, treatment with ES 30 and 90 mg/kg significantly decreased the AGEs levels compared with the diabetic model group (*P* < 0.05, *P* < 0.01, respectively). These results indicate that to some extent, ES is able to protect against AGEs level increase in diabetic rats.

### Effect of ES against STZ-induced diabetic renal histopathological changes in rats

**Fig. 7:**
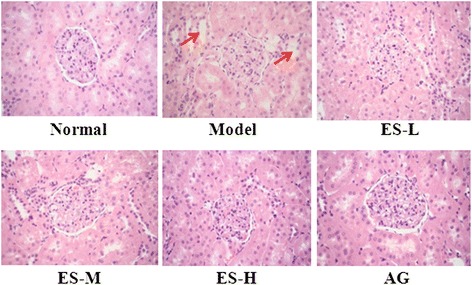
Effects of esculin on renal histopathological changes in the STZ-induced diabetes rats by HE staining. Representative photomicrographs of the renal in different groups. Magnification: ×200

HE staining was performed on renal sections to measure tubular damage. As shown in the representative pictures of renal sections, severe tubulointerstitial injuries including tubular epithelial cell detachments, cystic dilatation of tubules, and inflammatory cell infiltration occurred in kidneys of STZ-treated diabetic rats. However, the increased tubular damage in STZ-treated mice was reduced by treatment with esculin (Fig. 7).

### Effect of ES against STZ-induced AGEs accumulation in renal of rats

**Fig. 8:**
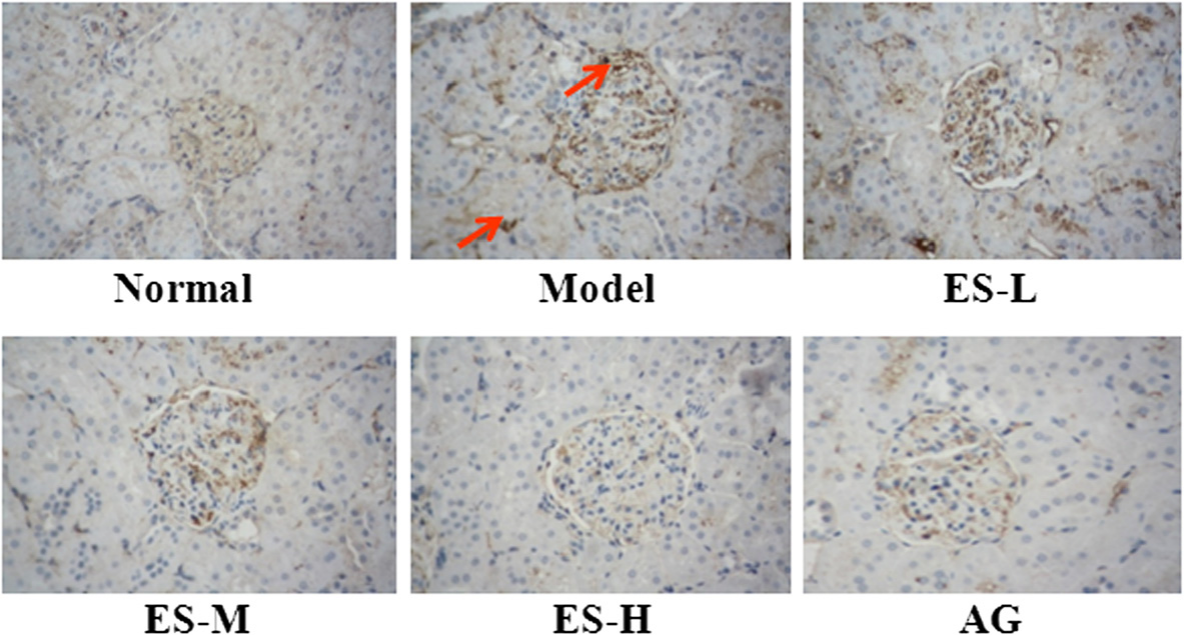
Effect of ES on renal AGEs accumulation in STZ-induced diabetic rats by immunochemistry. Representative photomicrographs of the renal in different groups. Magnification: ×200

Accumulation of AGEs is observed in kidney of diabetic rats, for which these molecules contribute to diabetes complications. In the present study, an increase of AGEs accumulation was found in the renal of the diabetic model group; however, treatment with ES 30 and 90 mg/kg resulted in obviously reduced AGEs accumulation (Fig. 8). These results indicate that ES is able to inhibit the nonenzymatic glycation of protein *in vivo* and may help to attenuate renal injury in diabetic rats.

## Discussion

Diabetes is increasing at epidemic proportions throughout the world. Most of the morbidity and mortality associated with diabetes results from macro and microvascular complications [12, 13]. Compared to macrovascular complications, microvascular complications, such as retinopathy, neuropathy, nephropathy, are much earlier and common in diabetes. Approximately 40 % of patients with diabetes may progress to nephropathy and a good metabolic control can prevent the development of diabetic renal injury [14]. Hyperglycaemia and dyslipidemia are two pathological characteristics of diabetes [15, 16]. Increased levels of glucose and lipids in serum and kidney can directly stimulate oxidative stress, inflammation, fibrosis and advanced glycation end products (AGEs) products formation in tubular and glomerular structures [17, 18].

Esculin, a coumarin derivative, was extracted from *Fraxinus rhynchophylla*, which belonging to a traditional medicinal plant from East Asia. Thuong PT, et al. [19] found that derivative coumarins fraxetin has direct protective properties against LDL oxidation at lower concentrations, and higher concentrations of fraxetin induce antioxidant enzymes via Nrf2/ARE activation. These effects suggest potential anti-atherosclerosis effects of Fraxinus rhynchophylla. Naaz F, et al. [20] demonstrated that the protective efficacy of esculin against pro-oxidant AFB1-induced nephrotoxicity in mice might be due to its antioxidants and free radical scavenging properties. Kang KS, et al. [11] found that esculin lessened the elevated blood creatinine levels in diabetic mice and ameliorated diabetes-induced renal dysfunction by reducing caspase-3 activation in the kidney. However, our results showed that ES could improve dyslipidemia, inflammation responses, renal damage in STZ-induced diabetic rats and the possible mechanism might be associated with the inhibition of AGEs formation. In this study, the results also showed that hyperglycaemia, dyslipidemia and systemic inflammatory responses were observed in STZ-induced diabetes rats. However, ES significantly improved dyslipidemia in diabetes, but did not significantly change the level of blood glucose.

Previous reports suggested that several humoral markers of inflammation are elevated in diabetic patients and inflammation is involved in the progression of diabetic nephropathy. Some pro-inflammatory cytokines, such as IL-6, IL-1β, and TNF-α are involved in matrix expansion and mesangial cells proliferation leading to glomerular fibrosis [21]. In the present study, our results showed that ES could dose-dependently attenuate the increase of IL-1, IL-6, ICAM-1 and NO levels in STZ-induced diabetic rats. These findings indicate that ES is able to ameliorate inflammation responses to a certain extent in diabetic rats.

Diabetic nephropathy is associated with mesangial cell expansion, thickening of glomerulra and tubular basement membrane, glomerulosclerosis and tubular necrosis. Previous reports suggested that the presence of hyperglycaemia and dyslipidemia causes functional and morphological kidney alterations that can induce renal dysfunction [15, 16]. Dyslipidemia can induce a glomerular phenotype characterized by proliferative and pro-inflammatory response, monocyte infiltration, increased extracellular matrix and podocytes degeneration [18]. It has been demonstrated that diabetes-induced oxidative stress and lipid alteration, in addition to a marked glucose elevation, play a detrimental role in the onset of nephropathy in diabetic animals [22, 23]. In the present study, it was shown that ES could obviously improve renal damage in STZ-induced diabetes rats.

Due to prolonged high glucose in diabetes, reducing sugars can react non-enzymatically with the amino groups to initiate a complex series of rearrangements and dehydrations, and then to produce a class of irreversibly cross-linked moieties termed AGEs [24]. The accumulation of AGEs is an important feature in the development of diabetic complications [25, 26]. The formation and accumulation of AGEs in various tissues have been shown to progress at an accelerated rate under hyperglycemic conditions [27, 28]. There is accumulating evidence that AGEs and receptor for AGEs interaction induces oxidative stress generation and subsequently evokes inflammatory reactions, thereby causing progressive alteration in renal architecture and loss of renal function in diabetes [29–31]. In particular, AGEs are thought to be involved in the pathogenesis of diabetic nephropathy via multifactorial mechanisms such as oxidative stress generation and overproduction of various growth factors and cytokines [32]. It is reported that AGEs have been recently shown to increase leukocyte adhesion to cultured retinal microvascular endothelial cells by inducing intracellular cell adhesion molecule-1 (ICAM-1) expression [33].AGEs modification of proteins may produce in changes charge, solubility, and conformation leading to molecular dysfunction as well as disrupting interactions with other proteins. AGEs also interact with specific receptors and binding proteins to influence the renal expression of growth factors and cytokines, implicated in the progression of diabetic renal disease [34].

Investigations by others also demonstrated that formation of AGEs is an important pathophysiological mechanism in the development of diabetic nephropathy [35]. Jung HA, et al. have found that esculin may play a crucial role in AGE inhibition and may be as potential candidates for use as therapeutic or preventive agents for diabetic complications [9]. In agreement with this, our research firstly demonstrated the inhibitory effect of ES on AGE formation in diabetic rats. The results showed that in serum of normal rats, the level of AGEs was very low, but in diabetic rats, it was significantly increased. However, the increase of AGEs level in serum was obviously depressed by the administration of ES and AG. In this present study, we also found that the accumulation of AGEs in renal, especially in glomerulra, was increased in STZ-induced diabetic rats, which was correlated with renal damage. Moreover, ES administration reduced the accumulation of AGEs in diabetic rats. These observations suggested that ES could restrain the AGEs accumulation in kidney. Therefore, these results suggest that AGEs accumulation might play a considerable role in the diabetic nephropathy and it is likely that inhibiting the formation of AGEs or removing established AGEs modifications will form an important component part of future therapy in patients with diabetes, acting in concert with conventional approaches to prevent diabetic renal injury.

## Conclusions

In conclusion, our research confirmed that ES had a protective effect against lipid metabolism disorders, inflammation responses, and renal injury in diabetic rats, the mechanism of which may be at least partly correlated with the amelioration of AGEs accumulation. Therefore, it can be concluded that ES may help to attenuate diabetes nephropathy.
